# Effect of resistance training and high-intensity interval training on metabolic parameters and serum level of Sirtuin1 in postmenopausal women with metabolic syndrome: a randomized controlled trial

**DOI:** 10.1186/s12944-023-01940-x

**Published:** 2023-10-19

**Authors:** Saeid Shamlou Kazemi, Ali Heidarianpour, Elnaz Shokri

**Affiliations:** Faculty of Sport Sciences, Bu-Ali Sina University, Hamedan, Iran

**Keywords:** Metabolic syndrome, High-intensity interval training, Resistance training, Sirtuin1

## Abstract

**Background:**

The present study analyzes the influence of resistance training (RT) and high-intensity interval training (HIIT) on metabolic indices and serum levels of Sirtuin1 (SIRT1) in postmenopausal women who suffer from the metabolic syndrome (MetS).

**Methods:**

45 postmenopausal women aged 45–65 years with MetS were divided into two intervention groups (RT and HIIT) and one control group, each consisting of 15 people. The RT group performed resistance training for both the upper and lower body, while the HIIT group completed 3 min(min) of high-intensity training at 80–90% of their maximum heart rate (HRmax), followed by moderate walking for 3 min at 55–65% of HRmax. These sessions were conducted for a duration of eight weeks and three times a week, with the samples being collected at the baseline and at the end of the treatment, i.e., week 8.

**Results:**

The results showed that weight, waist circumference, body mass index, fat mass, low-density lipoprotein, triglyceride, cholesterol, fasting blood sugar (FBS), hemoglobin A1c (HbA1C), systolic and diastolic blood pressure decreased, and SIRT1 increased significantly in both training groups. Systolic blood pressure, cholesterol, HbA1C, and FBS decreased more in the HIIT group. Skeletal muscle mass and 1-repetition maximum (1-RM) increased more in the RT group.

**Conclusions:**

RT and HIIT serve as one of the most effective strategies for therapeutically treating patients with metabolic syndrome.

**Trial registration:**

IRCT, IRCT20221120056548N1. Registered 23 November 2022 - Retrospectively registered.

## Introduction

In recent decades, metabolic syndrome (MetS) has become more prevalent, notably in less developed nations [1]. This disease is associated with high social and economic costs. It is defined as a set of interconnected factors that directly put individuals at a higher risk of getting coronary heart disease (CHD), other types of atherosclerotic cardiovascular disease (CVD), and type 2 diabetes (DMT2) [2]. Postmenopausal women undergo significant negative alterations in their body composition. Among the alterations are a reduction of muscle mass, an increase in fat mass, and fat redistribution to the central area of the body from the periphery. Therefore, there is a high risk for the occurrence of MetS in the postmenopausal period [3]. Sirtuin 1 (SIRT1) is one of the most important indicators in metabolic control and prevention of obesity and weight gain, and subsequent diseases such as metabolic syndrome, hypertension, type 2 diabetes, and heart diseases [4]. SIRT1 regulates important cellular processes such as apoptosis, insulin secretion, and metabolism. Sirtuin is a part of protein deacetylase that acts against oxidative stress and homeostasis control. In fact, by deacetylating histones, Sirtuins play a crucial role in various important functions, such as regulating the production of free radicals and lipid oxidation [5]. It has also been reported that the suppression of SIRT1 causes various complications, such as systemic inflammation and increased oxidative stress. This element acts as an effective factor in controlling overweight and metabolic components of the human body [6]. Consequently, there is a proposal to consider SIRT1 as a potentially effective therapeutic target for preventing and treating MetS. Its activation improves insulin resistance and glucose homeostasis [4].

The impact of exercise on boosting SIRT1 expression has been the main focus of numerous studies [4, 7]. The researchers believe that the reduction of glucose and the increase of nicotinamide adenine dinucleotide, due to exercise, causes the activation of SIRT1 [4]. Also, exercise and physical activity are recommended to control individual risk factors in people with MetS [8]. The consistent practice of physical activity can lead to weight loss, decreased blood pressure, and improved lipid profile by promoting the elevation of high density lipoprotein (HDL)-cholesterol and the decrease in triglycerides [9, 10].

Currently, various exercises are performed with different goals. Resistance training (RT) is a form of strength training exercise that focuses on gradually increasing the load on muscles to build strength. In this type of training, the muscles are required to exert force against an external load. It can be both safe and effective for older individuals [11]. RT has the potential to increase muscle mass and strength, improve insulin sensitivity, and enhance glucose oxidation [8]. Studies have been conducted on the effect of RT on MetS. It has been found through a study that adding RT as part of an exercise routine could potentially lower systolic pressure levels, stroke mortality, and heart disease mortality in those suffering from MetS [12]. According to Strasser’s meta-analysis, the application of RT has been proven to yield clinically and statistically significant results when it comes to mitigating MetS risk factors, specifically obesity, hemoglobinA1c (HbA1c) level, and systolic blood pressure. Therefore, it is a highly recommended approach in the treatment of type 2 diabetes and metabolic disorders [13]. Also, RT (three times a week) may have a positive impact on MetS Z-score in postmenopausal women by simultaneously reducing fasting glucose, improving body composition, and muscle strength [3].

On the other hand, High-intensity interval training (HIIT) has been proven to enhance insulin sensitivity, reduce blood pressure, and improve body composition in adults ranging from 20 to 77 years old [14]. HIIT, a specific form of cardiovascular training, includes alternating exercise and recovery phases with varying durations [15]. These exercises have recently received attention for their ability to induce positive health and performance adaptations [8]. Greeley et al.‘s study showed that HIIT can help reduce MetS risk factors, improve cardiovascular function, and optimize responsiveness to daily activities [16]. The popularity of HIIT training is partially due to the idea that it can be more successful in cutting abdominal and subcutaneous fat compared to other forms of exercise, which is an intriguing observation [17]. On the other hand, the maintenance of muscle health plays a crucial role in combating physical weakness and preserving metabolic well-being during one’s later years [18].

In total, despite the importance of the physiological role of SIRT1 in people with MetS, the response of this protein to RT and HIIT in patients with MetS is not yet well known. Also, it is not clear which of these exercises is more useful in postmenopausal women with MetS. Moreover, most of the studies on MetS have focused on diet and aerobic training, followed by pharmacological treatments. There is a lack of information about the impact of HIIT and RT training. In an effort to bridge this knowledge gap, this study will analyze the influence of HIIT and RT training on SIRT1 levels among postmenopausal women diagnosed with metabolic syndrome. In this specific group of women, this research will enhance the comprehension of the correlation between exercise and SIRT1 levels. Accordingly, the present clinical trial study seeks to answer the following questions: Do 8 weeks of HIIT and RT training affect circulating levels of SIRT1? Is HIIT exercise a more effective strategy in increasing SIRT1 and improving metabolic indices compared to RT?

## Methods

### Study design and participants

This study is a clinical trial (approved by the Iranian Registry of Clinical Trials, IRCT20221120056548N1 on 23 November 2022, retrospectively). It was conducted among women with MetS who were referred to the diabetes clinic of Imam Hossein Malayer Hospital due to diabetes between September and January 2021. The volunteer participants were briefed on the purpose of the study. They also filled in a written informed consent form. Approval was received from the Research Ethics Committee of Bu-Ali Sina University in Hamadan, Iran (IR.BASU.REC.1400.046), as the study observed the guidelines of the Declaration of Helsinki. A woman is classified as having metabolic syndrome depending on whether she presents with at least three out of five abnormal attributes as defined by the IDF standards (2006): these include (i) a waist measurement larger than or equal to 80 centimeters specifically for women; (ii) high blood pressure reaching above 130/85 mmHg; (iii) a fasting blood sugar (FBS) count higher than or equal to 100 mg/dL; (iv) elevated Triglyceride (TG) amounts of more than or equal to 150 mg/dL; and finally, (v) HDL- cholesterol < 50 mg/dL for women [19]. Women meeting these criteria were asked to take part in the present study.

The inclusion criteria included inactive postmenopausal women with an age range of 45–65 years who had no history of regular sports activity in the past year, and at least one year had passed since their last menstrual period. They also did not have any chronic musculoskeletal diseases and were non-smokers.

Exclusion criteria included: non-compliance with the study exercise protocol, any change in the patient’s routine program based on the physician’s opinion, decompensated heart failure, and the use of certain drugs or supplements. The individuals’ participation in the study was not disqualified due to their use of medications, as no alterations were made in terms of drug type or dosage. The subjects were also advised not to change their usual diet.

The G-Power software determined the sample size based on the previous results obtained for SIRT1 measured in humans [20]. With α = 0.05 and power (1-β) = 0.80, and effect size = 0.5, the participation of at least 42 subjects will be required. In total, 58 patients with metabolic syndrome were classified; 13 patients did not either have the inclusion criteria or were excluded from analysis for some other reason. Therefore, 45 patients remained in the study. They were randomly assigned into three groups: the HIIT training group (n = 15), the RT group (n = 15), and the control group (n = 15).

**Fig. 1:**
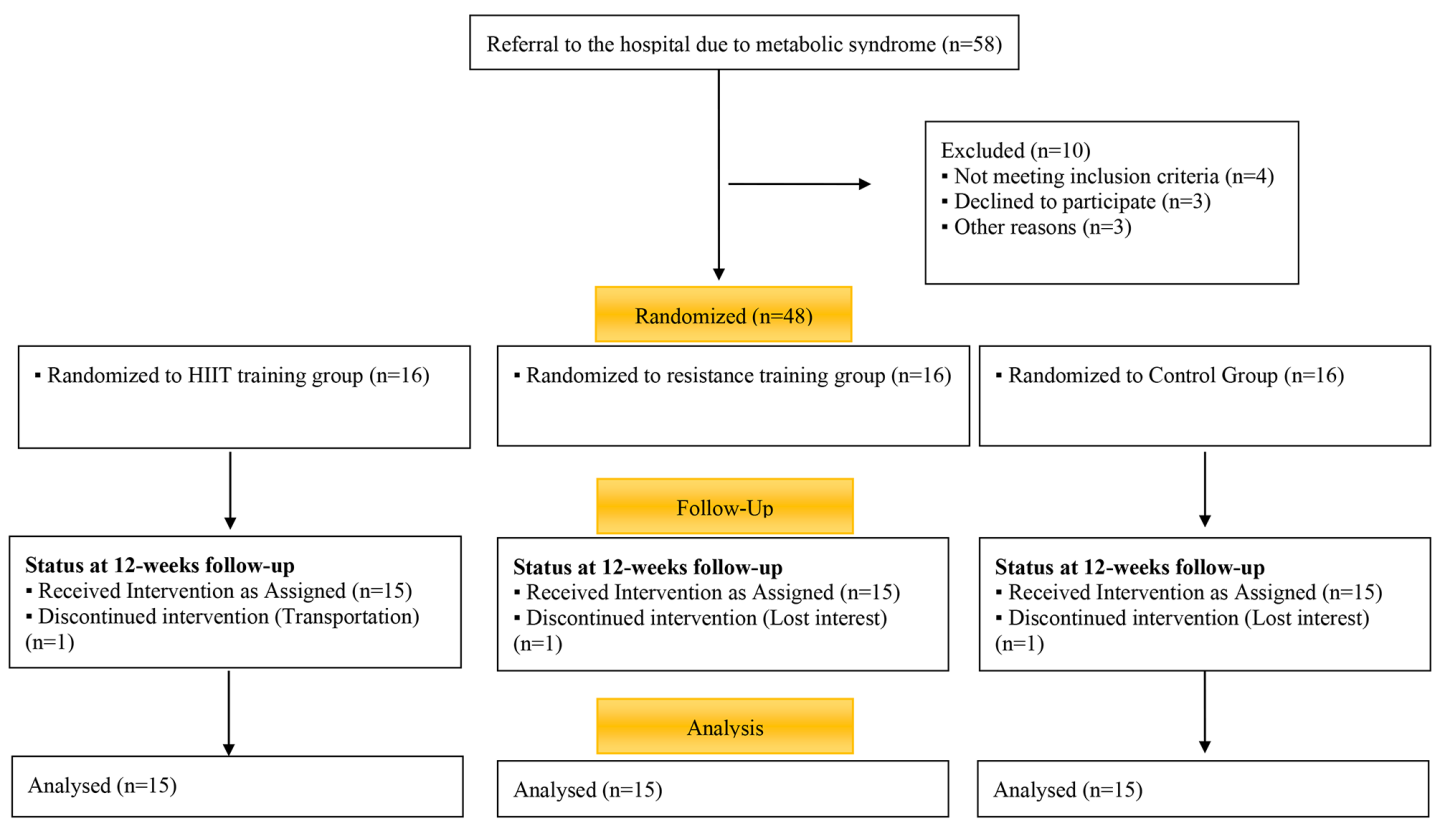
Flow diagram of study

### Exercise interventions

To familiarize the subjects, they were called to the gym 48 h before the program began. After a brief warm-up, the subjects were introduced to how to perform the exercises. Then they started practicing the exercises to familiarize themselves with the proper form and technique. The purpose of this familiarization period was to ensure the subjects understood how to correctly perform the exercises before the official start of the exercise program. This would help minimize errors and injuries during the actual program and allow the subjects to focus on implementing the exercises effectively. The training program for each group consisted of three training days per week for eight weeks (with 48 h of rest between each session). All training sessions started with a 10-minute(min) warm-up and stretching exercises, followed by a cool-down after finishing the exercises (During the final 5 min of each session, participants engaged in a cool-down period that included static movements targeting the larger muscle groups, lasting between 10 and 15 s per position and repeating each exercise 1–2 times). The exercise program was based on the recommendations of ACSM [8]. Two adept physical fitness trainers supervised the workout sessions. Furthermore, the experimental study instructed the control group to adhere to their usual routine throughout the duration of the investigation.

The HIIT group engaged in high-intensity interval aerobic exercise, alternating brisk walking and running with two active recovery periods at a moderate pace. The participants ran three times per session for three min. Those who could not run were instructed to walk as quickly as possible. The intensity of the exercise was set at 80–90% of maximum heart rate (HRmax). An active recovery period was implemented to interrupt the high-intensity period, during which participants walked at a moderate pace for three min at 55–65% of their HRmax. The HRmax was estimated using the equation ((208 – age) ∗ 0.7). Evaluation of the outcomes was conducted at baseline and after the 8-week intervention concluded [8].

The training program in the RT group consisted of two stages. In the first four weeks, the subjects performed various exercises such as leg press, chest press, front thigh, back thigh, side push, back lat, front arm, back arm pull, and two abdominal leg movements. Each exercise included three sets with eight to ten repetitions, and there was a 60-second rest period between sets. The intensity of the exercises was set at 75% of the subjects’ 1-repetition maximum (1-RM). After four weeks, the training program was modified. The subjects now performed eight repetitions of each exercise, with a rest period of 90 s between the sets. The intensity was increased to 80% of their 1RM. The resistance exercises followed a linear progression and the classic model, which involved starting with higher volume and lower intensity, and then gradually increasing the intensity while decreasing the volume [3].

All exercises included in the study were evaluated for muscle strength using a one-repetition maximum test. Each individual was required to perform eight repetitions using only 50% of their one-repetition maximum, as determined by personal assessment. After a brief pause, they performed an additional three repetitions at increased intensity (70% of their one-repetition maximum). Following a 3-min rest, succeeding trials were executed with increased weights for one repetition until the highest weight lifted in a single effort was identified within three attempts, with interludes of 3–5 min between each trial [3].

### Body composition

The weight of subjects was measured using a SECA scale (Germany, Hamburg) with an accuracy of 0.5 kg, and a tape measure mounted on the wall (accuracy = 0.1 cm) was applied to measure the height of the subjects. Body mass index (BMI) was also calculated as weight in kg/height in m^2^. Using a tape measure, the waist circumference (WC) was measured from the lowest point between the pelvic bone and the last rib. The waist-to-hip circumference ratio (WHR) was measured according to the equation of waist circumference/hip circumference (WHR = waist circumference/hip circumference). The Dezenberg et al.‘s (1999) formula was used to estimate fat mass (FM) [21], which has already been validated in the elderly [22]. The Lee et al. equation was utilized to estimate skeletal muscle mass (SMM) [23], which has also been validated in the elderly [24]. To measure blood pressure, a digital arm sphygmomanometer (OMRON, Japan) was applied while participants remained stationary in their chairs. To minimize the margin of error, measurements were conducted twice. The watch (Polar S610, Finland, Kempele) fastened to the wrist was used to measure heart rate (HR) [25]. According to the protocols stated by the American Thoracic Society, the 6-minute walk test (6MWT) was used to measure maximal oxygen uptake (VO2peak). Under the given time frame, participants were guided to walk along a 30-meter line with an interval of 1.5 m in an outdoor corridor for a duration of 6 min, where their distance was noted in meters. Patients were urged to complete the walking tests at their fastest speed, in hallways or outdoor corridors. The test has been given and confirmed on senior female individuals [26].

### Biochemical blood analysis

Blood Sampling was conducted in two stages. The first sample was taken one day before the familiarization session with training, and the second one was taken 48 h after the last training session in the eighth week. During both phases and after a 12-hour fast (excluding water), test specimens were obtained from 8:30 until 9:00 a.m. Before each stage of sampling, the participants were in a sitting position resting for a few min; following their rest, 10 mL of blood was obtained from their brachial vein in their elbow area in the shortest possible time. Finally, after blood collection, the samples were placed at room temperature for 20 min to clot, and subsequently, the tubes containing the samples were centrifuged for 12 min at 3,000 revolutions per min (RPM). Then, after separation, the serum was stored at -80 ˚C. Another part was prepared as plasma with the addition of EDTA anticoagulant in order to measure the blood glucose level and HbA1c level. The glycated N-terminal valine of the β-chain is the basis for measuring HbA1c concentration through a particular chemical reaction [27]. Blood lipids (triglyceride, total cholesterol, low-density lipoprotein (LDL)-cholesterol, and HDL-cholesterol) were measured enzymatically. Serum SIRT1 concentration was measured using the ELISA kit (Zell Bio Co, Germany), with a normal range of 5–160 ng/ml and sensitivity of 1 ng/ml [28].

### Data analysis

The assessment of data normality involved using the Shapiro-Wilk test. The data is reported as mean ± standard deviations (SDs). Furthermore, the data was analyzed using a 2-way [3 (group) × 2 (time)] ANOVA on the SPSS statistical software (version 26). Differences in the measures were detected by using a Bonferroni post hoc test when a significant F-value was attained. Statistical significance was set at less than 0.05.

## Results

### Anthropometric and clinical parameters and symptoms of metabolic syndrome

The participants’ anthropometric and clinical parameters in the three groups (HIIT, RT, and control) and two stages (baseline and 8 weeks) are presented in Table 1. The data distribution was normal. Table 1 shows no meaningful differences between the three groups in any of the parameters during the baseline stage. Additionally, Table 1 shows that weight, BMI, WC, WHR, FM had a significant decrease and Vo2peak had a significant increase in both training groups (*P* < 0.05). Table 2 shows the symptoms of metabolic syndrome. Table 2 confirms that LDL-cholesterol, TG, cholesterol, systolic blood pressure (SBP), diastolic blood pressure (DBP), FBS, and HbA1C had a significant decrease and HDL-cholesterol, SIRT1 had a significant increase in both training groups (*P* < 0.05). Observations made during the study revealed no significant variations within the control group (*P* > 0.05). A significant increase was witnessed in SMM and 1-RM only in the RT group. The findings also proved that cholesterol, SBP, and FBS decreased more in the HIIT group rather than the RT group (*P* < 0.05). Also, Vo2peak increased more in the HIIT group than in the RT group (*P* < 0.05). No meaningful difference was found between the effects of the two exercise programs on the SIRT1 levels (*P* > 0.05).

**Table 1:** Subject’s physiological and anthropometric characteristics in two stage (Baseline, 8 weeks) and three groups (HIIT, RT, Control)

Parameters	HIIT		RT		Control	
	baseline	8 weeks	Baseline	8 weeks	baseline	8 weeks
Weight (kg)	72.75 ± 3.19	**67.1 ± 3.31** ^**ab**^	71.33 ± 3.97	**68 ± 4.16** ^**ab**^	70.5 ± 2.54	70.55 ± 2.79
Height (cm)	162.47 ± 8.53	162.68 ± 8.53	161.94 ± 8.12	162.1 ± 8.32	161.73 ± 7.1	161.79 ± 7.21
BMI (kg/m^2^)	28.33 ± 0.36	**26.04 ± .44** ^**ab**^	27.94 ± 0.71	**25.80 ± 1.00** ^**ab**^	27.14 ± 0.38	27.17 ± 0.52
WC (cm)	99.12 ± 11.4	**95.46 ± 10.51** ^**ab**^	98.48 ± 10.22	**95.01 ± 10.74** ^**ab**^	98.53 ± 11.6	99.27 ± 11.86
WHR	0.93 ± 0.02	**0.88 ± .01** ^**ab**^	0.91 ± 0.02	**0.89 ± .01** ^**ab**^	0.91 ± 0.01	0.91 ± 0.02
FM (kg)	28.35 ± 5.69	**25.11 ± 4.43** ^**ab**^	27.63 ± 6.02	**26.52 ± 5.91** ^**ab**^	27.56 ± 5.39	27.88 ± 7.13
SMM (kg)	18.74 ± 3.30	18.25 ± 5.39 ^**c**^	17.76 ± 2.64	**19.62 ± 5.14** ^**ab**^	17.66 ± 3.51	17.82 ± 3.44
1-RM (kg)	25.45 ± 11.52	28.72 ± 12.11 ^**c**^	23.48 ± 10.19	**38.61 ± 13.46** ^**ab**^	22.71 ± 12.66	24.37 ± 10.08
6MWT (m)	468 ± 55.78	**865 ± 62.72** ^**abc**^	504 ± 79.58	**650 ± 81.39** ^**ab**^	430 ± 68.40	419 ± 77.26
Vo_2_peak (ml.kg^− 1^.min^− 1^)	22.94 ± 8.8	**27.15 ± 7.2** ^**abc**^	23.04 ± 9.11	**25 ± 6.53** ^**ab**^	22.57 ± 8.49	22.31 ± 8.66

Data presented are the mean ± standard deviation. *P* < 0.05. a: significantly different between baseline stage and 8-weeks, b: Significant difference with the control group, c: Significant difference with the RT group, (HIIT: high-intensity interval training, RT: resistance training, BMI: Body mass index, WC: waist circumference, WHR: Waist-Hip Ratio, FM: Fat mass, SMM: Skeletal muscle mass, 1-RM: 1-repetition maximum, 6MWT: 6-Minute Walk Test,Vo_2_ peak: Peak oxygen uptake)

**Table 2:** Subject’s metabolic syndrome symptoms in two stage (Baseline, 8 weeks) and three groups (HIIT, RT, Control)

Parameters	HIIT		RT		Control	
	baseline	8 weeks	Baseline	8 weeks	baseline	8 weeks
HDL-C (mg/dl)	51.9 ± 4.60	**64.7 ± 5.22** ^**ab**^	50.2 ± 3.39	**62.7 ± 4.47** ^**ab**^	52.5 ± 3.71	51 ± 5.22
LDL-C (mg/dl)	116.8 ± 19.79	**85.7 ± 15.47** ^**ab**^	105.5 ± 23.38	**86.8 ± 13.81** ^**ab**^	108.9 ± 18.74	113.4 ± 20.75
TG (mg/dl)	193.8 ± 15.73	**169.6 ± 19.58** ^**ab**^	192.4 ± 19.29	**171.9 ± 16.33** ^**ab**^	194.9 ± 11.53	200.6 ± 21.42
Cholesterol (mg/dl)	207.4 ± 12.78	**180.6 ± 17.36** ^**abc**^	205.8 ± 9.06	**188.8 ± 9.06** ^**ab**^	199.7 ± 12.11	201.5 ± 15.68
SBP (mmHg)	157 ± 4.89	**147.9 ± 4.21** ^**abc**^	160 ± 3.33	**153.2 ± 5.09** ^**ab**^	154.4 ± 5.10	155.2 ± 6.23
DBP (mmHg)	103.7 ± 4.6	**95.1 ± 3.62** ^**ab**^	100.4 ± 4.35	**98.8 ± 5.55** ^**ab**^	101.8 ± 3.06	103.4 ± 7.16
FBS (mg/dl)	151.4 ± 2.83	**145.4 ± 3.24** ^**abc**^	148.3 ± 4.49	**144.3 ± 3.22** ^**ab**^	144.9 ± 4.97	145.8 ± 7.88
HbA1c (%)	8.8 ± 0.71	**7.55 ± .77** ^**abc**^	8.75 ± 1.03	**7.9 ± .43** ^**ab**^	8.25 ± 0.54	8.6 ± 0.84
SIRT1 (ng/ml)	24.32 ± 5.41	**28.19 ± 8.27** ^**ab**^	23.52 ± 3.75	**27.42 ± 7.83** ^**ab**^	22.75 ± 6.09	21.11 ± 5.75

Data presented are the mean ± standard deviation. *P* < 0.05. a: significantly different between baseline stage and 8-weeks, b: Significant difference with the control group, c: Significant difference with the RT group, (HIIT: high-intensity interval training, RT: resistance training, HDL-C: High density lipoprotein-cholesterol, LDL-C: Low density lipoprotein-cholesterol, TG: triglyceride, SBP: systolic blood pressure, DBP: diastolic blood pressure, FBS: fasting blood sugar, HbA1c: hemoglobin A1c, SIRT1: sirtuin1)

In addition, the results revealed that following 8 weeks of HIIT and RT exercises, all variables decreased significantly in comparison to the control group (except HDL-cholesterol, Vo2peak, and SIRT1, which significantly increased) (*P* < 0.05).

### Changes in MetS symptoms

**Fig. 2:**
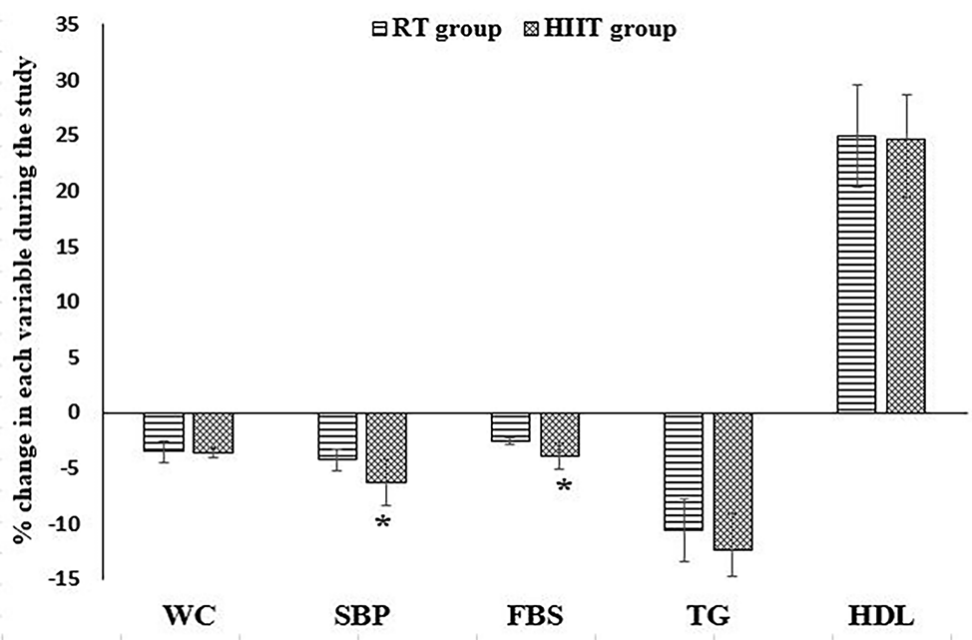
Comparison of changes in symptoms of metabolic syndrome during the study in two groups of HIIT and RT (*: significantly different between HIIT and RT groups, HIIT: high-intensity interval training, RT: resistance training, WC: waist circumference, SBP: systolic blood pressure, FBS: fasting blood sugar, TG: triglyceride, HDL-C: high-density lipoprotein-cholesterol)

Figure 2 shows the changes in metabolic syndrome symptoms as a result of HIIT and RT exercises. As it is obvious, there was a significantly higher reduction of SBP and FBS in the HIIT group compared to the RT group.

### Correlation analysis

Table 3 displays the Pearson correlation between changes in parameters during the baseline stage and after 8 weeks of HIIT training. As shown in Table 3, changes in SIRT1 after HIIT training had a significant negative correlation with changes in BMI, FM, FBS, and HbA1c.

**Table 3:** Correlation between parameter changes in the baseline stage and after 8 weeks HIIT training

Parameters	Δ FM (kg)		Δ SMM (kg)		Δ Vo_2_peak (ml.kg^− 1^.min^− 1^)		Δ FBS (mg/dl)		Δ HbA1c (%)		Δ SIRT1 (ng/ml)	
	r	*P*	r	*P*	r	*P*	r	*P*	r	*P*	r	*P*
Δ BMI (kg/m^2^)	0.74	0.001*	0.05	0.88	-0.26	0.05 *	0.28	0.05*	0.04	0.39	-0.38	0.001*
Δ FM (kg)			0.09	0.31	-0.24	0.03 *	0.23	0.58	0.10	0.61	-0.32	0.03*
Δ SMM ((kg)					0.05	0.22	0.14	0.64	0.002	0.77	0.27	0.66
Δ Vo_2_peak (ml.kg^− 1^.min^− 1^)							0.05	0.21	-0.11	0.38	0.15	0.91
Δ FBS (mg/dl)									0.27	0.02*	-0.19	0.04*
Δ HbA1c (%)											-0.24	0.01*
Δ SIRT1												

Δ: value at 8 weeks − value at baseline, BMI: Body mass index, FM: Fat mass, SMM: Skeletal muscle mass, Vo_2_ peak: Peak oxygen uptake, FBS: Fasting blood sugar, HbA1c: hemoglobin A1c, SIRT1: sirtuin1. *: Significant correlation

Also, Table 4 displays the Pearson correlation between changes in parameters during the baseline stage and after 8 weeks of resistance training (RT). It proves that changes in SIRT1 are negatively and significantly correlated with changes in BMI and FM, FBS, and HbA1c.

**Table 4:** Correlation between parameter changes in the baseline stage and after 8 weeks RT

Parameters	Δ FM (kg)		Δ SMM (kg)		Δ Vo_2_peak (ml.kg^− 1^.min^− 1^)		Δ FBS (mg/dl)		Δ HbA1c (%)		Δ SIRT1 (ng/ml)	
	r	*P*	r	*P*	r	*P*	r	*P*	r	*P*	r	*P*
Δ BMI (kg/m^2^)	0.68	0.002*	-0.37	0.01*	-0.14	0.92	0.19	0.03*	0.22	0.09	-0.25	0.04*
Δ FM (kg)			-0.11	0.48	-0.24	0.03	0.37	0.4	0.17	0.31	-0.28	0.01*
Δ SMM ((kg)					0.36	0.07	0.08	0.25	-0.13	0.06	0.07	0.08
Δ Vo_2_peak (ml.kg^− 1^.min^− 1^)							0.12	0.08	-0.07	0.08	0.23	0.11
Δ FBS (mg/dl)									0.22	0.56	-0.14	0.01*
Δ HbA1c (%)											-0.33	0.01*
Δ SIRT1												

Δ: value at 8 weeks − value at baseline, BMI: Body mass index, FM: Fat mass, SMM: Skeletal muscle mass, Vo_2_ peak: Peak oxygen uptake, FBS: Fasting blood sugar, HbA1c: hemoglobin A1c, SIRT1: sirtuin1. *: Significant correlation

## Discussion

The findings of the present study imply that 8 weeks of RT and HIIT positively affected the metabolic profile of the participants with MetS compared to the controls. This short-term period of RT and HIIT, along with the reduction of cardio-metabolic risk factors, improved SIRT1 levels in women with MetS. No meaningful difference was discovered between the two exercise groups in their SIRT1 levels. However, HIIT was more suitable than RT in improving cardiorespiratory fitness (Vo2peak) and reducing systolic blood pressure, cholesterol, Hb1AC, and FBS. On the other hand, RT was more effective in increasing muscle mass and strength.

Studies on metabolic disorders have sparked interest in the role of mammalian SIRT1 protein (encoded by the SIRT1 gene), as it participates in critical physiological functions such as glucose metabolism and lipid breakdown [29]. Few studies have investigated the effect of different types of physical activity on SIRT1 serum levels. For instance, Ferrara et al. reported that six weeks of HIIT training increased the activity level of SIRT1 [30]. Studies have revealed that exercise leads to elevated activity and protein levels of SIRT1 [31]. Another study conducted by Jia et al. reported that continuous and moderate-intensity exercise training can increase the level of SIRT1 in people with heart disease [32]. Contrary to the findings of this research, Ma et al. showed that four weeks of high-intensity exercise training did not affect SIRT1 levels in healthy men [33], and Marton et al. indicated that exercise training did not significantly affect SIRT1 serum levels [34]. The difference between the findings of this research and other investigations could possibly be attributed to the variances in the approach deployed, age of the subjects, gender, physical condition, and the type of recommended exercise. In general, it can be stated that the evidence consistent with the present research suggests that regular exercise increases the serum level of SIRT1. Hence, a part of the positive effects of physical activity on anthropometric/metabolic parameters is attributed to the role of this protein. A correlation between SIRT1 and positive insulin secretion regulation in pancreatic β-cells has been documented in previous studies [35]. Moreover, under insulin-resistant conditions, enhanced expression of SIRT1 led to an improvement in insulin sensitivity [36]. In this respect, the results of the present research were indicative of a significant negative correlation between SIRT1 changes after 8 weeks of both HIIT and RT training and HbA1c changes. Also, Evidence suggests that during exercise, AMP-dependent protein kinase and nicotinamide phosphoribosyl transferase increase the intracellular level of NAD+. Its increase is associated with the stimulation and increase of SIRT1, which ultimately leads to fat oxidation, glucose absorption, and mitochondrial biogenesis through deacetylation of alpha and gamma receptor activated by peroxisome proliferator [37]. In addition, it is reported that SIRT1 has the ability to enhance mitochondrial biogenesis and fatty acid oxidation with the assistance of peroxisome proliferator-activated receptor γ coactivator-1α (PGC-1α). It activates PGC-1α through deacetylation directly [38]. Therefore, the increase of SIRT1 probably played a role in the reduction of weight, cholesterol, triglycerides, and fat mass in the current study. However, this claim has not been directly measured.

Dyslipidemia and cardiovascular disease risk factors are common among patients with MetS. The results of the current study suggest that regular physical activity can improve dyslipidemia and cardiovascular risk factors, in line with the findings of other studies [9, 39].

Moreover, Cuddy et al., after investigating the effect of HIIT exercises on cardiometabolic health indicators of a group of obese middle-aged men and women, reached the conclusion that HIIT exercises have effective improvements on MetS indicators [40]. Similar to the findings of this research, Zaer Ghodsi et al. stated that HIIT exercises have a positive effect on body composition indicators and lipid profile of women, and therefore can cause an improvement in lipid profile (decrease of LDL-cholesterol and cholesterol, and increase of HDL-cholesterol) [39]. In research conducted by Normandin et al., the results showed that RT has no significant effect on MetS indicators except for changes in body weight [41]. On the other hand, Dieli-Conwright et al. reported that RT along with aerobics improves MetS parameters in cancer patients [42]. Exercise activates the adenylate cycle by increasing the activity of epinephrine and glucagon, which is followed by an increase in the adenosine monophosphate cycle. This causes the activity of hormone-sensitive Lipoprotein lipase (LPL) to provide free TGs as an energy source. The activation of AMP kinase stimulates the oxidation of fatty acids, uptake of glucose, and the biogenesis of mitochondria. Also, exercise causes the rapid absorption of free fatty acids from the blood circulation. Fat oxidation provides strong evidence for the adaptive role of exercise in improving blood lipid profile by increasing HDL-cholesterol levels and reducing LDL-cholesterol levels and triglycerides. Sports activities increase the enzyme lipoprotein lipase and lecithin cholesterol acyltransferase (LCAT), and these two enzymes decrease LDL-cholesterol, triglycerides, cholesterol, and increase HDL-cholesterol [43]. Also, it has been stated that improving the oxidation capacity of fatty acids and lipid profile following HIIT programs is in close association with increased expression of key metabolic enzymes in mitochondria and skeletal muscles. This includes a significant increase in the expression of 3-hydroxyacyl-coa dehydrogenase, citrate synthase, muscle beta-hydroxyacyl-coa dehydrogenase, and fatty acid-binding protein [44].

A major contributor to MetS, insulin resistance is correlated with a greater risk for cardiovascular disease and mortality [45]. Sedentary lifestyle and improper diet are also correlated with insulin resistance, and the change of this parameter is more noticeable in elderly women after menopause [46]. Nevertheless, it has been confirmed that sports activity along with a healthy lifestyle cause insulin sensitivity, and intense periodic training through different pathways reduces blood glucose and improves insulin sensitivity. In this regard, HIIT causes glucose absorption in skeletal muscles, increases Glut4 content, and increases insulin sensitivity through the depletion of intramuscular glycogen [47]. In addition, it has been reported that sports activities increase mitochondrial biogenesis in the muscles of patients with MetS [48] and improve the phosphorylation and activation of insulin receptors by insulin in muscle and fat tissue. Also, exercise improves insulin function in muscles by reducing intracellular accumulation of triglycerides and increasing fatty acid oxidation [49]. Resistance exercise is a viable solution for enhancing insulin sensitivity as it promotes growth and maintenance of lean body mass, leading to an increase in glucose storage and elimination from circulation. This ultimately requires less insulin in obese adults who aim to maintain their normal glucose tolerance levels [42]. Regarding the application of RT on HbA_1_C, the results revealed that the training program had a significant effect on reducing the amount of HbA_1_C and FBS. In fact, RT produces favorable changes in insulin sensitivity by increasing skeletal muscle mass, glucose storage, increasing glucose clearance from blood circulation, and improving mitochondrial oxidative capacity [50]. In a meta-regression analysis study, it was found that a decrease in HbA1c and insulin after RT was correlated with exercise intensity [51]. Exercise intensity is a crucial factor in regulating insulin sensitivity. Physiologically, lower exercise intensity limits the capacity of the mitochondrial oxidative enzyme, subsequently leading to a decrease in glucose absorption by skeletal muscles and an increase in plasma glucose [8]. These harmful effects reduce insulin sensitivity and increase glucose deposition in fat cells.

One of the major factors that puts individuals at risk of heart disease is high blood pressure [12]. Prevention and non-pharmacological treatment of arterial hypertension include changes in lifestyle, such as physical activity to control blood pressure levels. Physical activity can help lower blood pressure by greatly reducing stress, even after an exercise session, due to a phenomenon called post-exercise hypotension. Aerobic and strength exercises are also associated with a considerable improvement in maintaining and reducing blood pressure levels [52]. It is believed that the reduction of blood pressure following physical activity is due to the reduction of peripheral vascular resistance, which is achieved through hormonal and structural neuro reactions with reduced sympathetic nerve activity and increased diameter of the arterial lumen. Various mechanisms have been suggested to lower blood pressure, such as improvements in inflammation, oxidative stress, endothelial function, body mass, arterial compliance, activity of the renin-angiotensin system, renal function, parasympathetic activity, and sensitivity to insulin. Exercise can elicit a wide range of antihypertensive responses, which are highly variable. Differences in exercise models, environmental factors, and genetic factors may be the cause of significant changes in blood pressure response to exercise [53].

According to the obtained results, the type, intensity, and duration of the exercise program are effective in influencing lipid factors. RT is a powerful anti-aging tool that combats the age-related reduction of myosin heavy chain gene transcripts. It boosts synthesis rate of muscle protein, enhances muscular mass quality and functionality, and promotes better glucose oxidation and insulin sensitivity [8]. Therefore, in order to examine the benefits of the two different exercise types on metabolic outcomes in this research, a randomized trial was designed for eight weeks. Following the intervention, the results of this research showed that the eight-week exercise positively affected the metabolic profile of those with MetS. However, In the present study, the upstream and downstream factors of SIRT1 such as AMPK, FOXO3 and NF-KB have not been measured to accurately determine the signaling pathway of HIIT and resistance exercises in weight control and other parameters. Therefore, it is recommended to measure the activity of SIRT1 in moderate-intensity continuous training (MICT) and low intensity continuous training (LICT) activities in addition to measuring the mentioned indicators in future research.

### Strengths and limitations

There were certain strengths found in this study. By utilizing random patient sampling for the formation of study groups, the probability of biases or distortions arising from patient assignment is minimized. By using a control group and experimental groups, the comparison of outcomes and evaluation of treatment efficacy becomes possible. In addition, this study showed the potential role of Sirt1-mediated lipid oxidation in the improvement of metabolic syndrome symptoms.

There were certain limitations associated with the current investigation. In the present study, considering the fact that the study was cross-sectional, a small number of samples, lack of recording of the food intake of the subjects, and drinking habits, more studies are required to provide a definitive recommendation.

## Conclusion

The findings point to the conclusion that regular HIIT and resistance exercise are likely to improve the status of serum levels of SIRT1 in postmenopausal women with metabolic syndrome. HIIT training can more effectively reduce the symptoms of MetS, but RT is more suitable for improving muscle mass and strength. Therefore, from a clinical point of view, it is recommended that postmenopausal women with metabolic syndrome include regular exercise in their life plan and participate in a combination of HIIT and RT.

## Data Availability

The datasets generated and/or analyzed during the current study will be available from the corresponding author upon reasonable request.

## List of Abbreviations

RT: Resistance training
HIIT: High-intensity interval training
SIRT1: Sirtuin1
MetS: Metabolic syndrome
HRmax: Maximum heart rate
FBS: Fasting blood sugar
HbA1C: Hemoglobina1c
1-RM: 1-repetition maximum
CHD: Coronary heart disease
CVD: Cardiovascular disease
DMT2: Type 2 diabetes
HDL: High density lipoprotein
LDL: Low density lipoprotein
TG: Triglyceride
BMI: Body mass index
WC: Waist circumference
WHR: waist circumference/hip circumference
FM: Fat mass
SMM: Skeletal muscle mass
HR: Heart rate
6MWT: 6-minute walk test
Vo2peak: Peak oxygen uptake
RPM: Revolutions per min
ANOVA: Analysis of variance
SBP: Systolic blood pressure
DBP: Diastolic blood pressure
PGC-1α: Peroxisome proliferator-activated receptor γ coactivator-1α
LPL: Lipoprotein lipase
LCAT: Lecithin cholesterol acyltransferase
MICT: Moderate-intensity continuous training
LICT: Low intensity continuous training
